# Electrical Conduction Mechanism and Dielectric Properties of Spherical Shaped Fe_3_O_4_ Nanoparticles Synthesized by Co-Precipitation Method

**DOI:** 10.3390/ma11050735

**Published:** 2018-05-05

**Authors:** Adrian Radoń, Dariusz Łukowiec, Marek Kremzer, Jarosław Mikuła, Patryk Włodarczyk

**Affiliations:** 1 Faculty of Mechanical Engineering, Silesian University of Technology, Konarskiego 18 a St., 44-100 Gliwice, Poland; adrian-radon@wp.pl (A.R.); marek.kremzer@polsl.pl (M.K.); jaroslaw.mikula@polsl.pl (J.M.); 2 Institute of Non-Ferrous Metals, ul. Sowinskiego 5, 44-100 Gliwice, Poland; patrykw@imn.gliwice.pl

**Keywords:** magnetite nanoparticles, electrical conduction mechanism, Koop’s theory, Jonscher’s power law, correlated barrier hopping, non-overlapping small polaron tunneling

## Abstract

On the basis of dielectric measurements performed in a wide temperature range (173–373 K), a comprehensive analysis of the dielectric and electrical properties of magnetite nanoparticles electrical conduction mechanism of compressed spherical shaped Fe_3_O_4_ nanoparticles was proposed. The electrical conductivity of Fe_3_O_4_ nanoparticles was related to two different mechanisms (correlated barrier hopping and non-overlapping small polaron tunneling mechanisms); the transition between them was smooth. Additionally, role of grains and grain boundaries with charge carrier mobility and with observed hopping mechanism was described in detail. It has been confirmed that conductivity dispersion (as a function of frequencies) is closely related to both the long-range mobility (conduction mechanism associated with grain boundaries) and to the short-range mobility (conduction mechanism associated with grains). Calculated electron mobility increases with temperature, which is related to the decreasing value of hopping energy for the tunneling of small polarons. The opposite scenario was observed for the value of electron hopping energy.

## 1. Introduction

Many different ferrites containing divalent, trivalent and quadrivalent ions were synthesized [1]. Interest in ferrites is connected especially with their magnetic and electrical properties, as well as their microwave absorption and photocatalytic properties. For example, Li et al. prepared MnZnFe_2_O_4_ nanoparticles (with chemical formula Mn_0.8_Zn_0.2_Fe_2_O_4_) with superparamagnetic behavior, i.e. high magnetization (79.6 emu/g), and small size (15–20 nm) [2]. Recently, the dielectric properties, electrical conductivity and microwave absorption properties of ferrites are the focus of our research. This phenomenon is associated with the possibility of using ferrites as electromagnetic interference shielding materials (especially as microwave absorbers), which is related to the their high reflection loss [3,4]. Ferrites can be also applied as inductors, magnetic field sensors, switched-mode power supplies, and ferromagnetic insulators in electrical circuits [5]. Magnetite nanoparticles (Fe_3_O_4_ NPs) are among the most popular nanoferrites. The co-precipitation method is a simple synthesis method, in which low cost subtracts can be used to obtain spherical shaped nanoparticles [6,7]. Interest in Fe_3_O_4_ NPs is associated not only with their superparamagnetic properties [8,9], but also with their highly reactive surface. Moreover, various nanoparticles can be deposited on ferrites [10,11]. The effect of substitution of ions on dielectric properties and electrical conductivity has already been described for MnFe_2_O_4_ (with praseodymium addition) [12]. Additionally, the influence of the shape of nanoparticles on dielectric and microwave absorption properties was described for nanorods and spherical shaped Fe_3_O_4_ NPs by Jazirehpour and Seyyed Ebrahimi. They reported that spherical shaped magnetite nanoparticles had the highest value of reflection loss [13]. The influence of cation distribution between octahedral and tetrahedral sites on the physical properties of ferrites has also been studied in detail [14]. The conduction mechanism of different ferrites and their dielectric properties were analyzed according to three models: Maxwell-Wagner model, Koop’s phenomenological theory and the Jonscher’s power law [12]. Koop’s theory was used to describe relationship between frequency and AC conductivity for different ferrites. According to this theory, in a low frequency region, grain boundaries with high resistance are responsible for constant conductivity, whereas in a high frequency region, the increase of the AC conductivity value is associated with grains with much higher conductivity than grain boundaries [15]. Maxwell-Wagner model is generally used to describe the polarization of grain boundaries in the low frequency region, and is applied to all inhomogeneous materials, such as multiphase materials and materials with large amount of interfaces and defects [16,17,18]. Jonscher’s power law is used to describe the electrical conductivity of many types of disordered solids. According to the *s*(*T*) behavior, conduction mechanism can be identified [19,20,21]. Different conduction mechanisms as non-overlapping small polaron tunneling (NSPT), overlapping large polaron tunneling (OLPT), quantum mechanical tunneling (QMT), and correlated barrier hopping (CBH) models were proposed for different *s*(*T*) behaviors [22,23,24,25]. Moreover, one material can be characterized by two or more different conduction mechanisms in different temperature ranges [26].

However, studies in which electrical conductivity of different samples was analyzed using models associated with microstructure (Koop’s, Maxwell-Wagner) and with dispersion of AC conductivity (Jonscher’s power law, *s*(*T*) behavior) are rare. Most of the literature only confirm that Jonscher’s power law can be used to describe the electrical conductivity of ferrites [27]. In their work, Velhal et al. showed that electrical conduction can be related to small polarons in cobalt ferrite nanoparticles; however, this result has not been combined with the previously described electrical conduction mechanism associated with hopping of electrons, and has not been confirmed by an analysis of *s*(*T*) behavior [28]. Moreover, many works only attributed variations of AC conductivity with frequency to the hopping of electrons between Fe^3+^ and Fe^2+^ ions and conduction by small polarons, without using the Jonscher’s power law, and determining the conduction mechanism model or hopping energy value [15,16,29]. Based on our knowledge, this study presents, for the first time, a clear connection between models associated with microstructure and with dispersion of AC conductivity. The combination of these two approaches can avoid some inaccuracies related to the description of electrical properties. For example, Panda et al. have shown, that the OLPT model can be used to describe the AC electrical conductivity of cobalt ferrite nanoparticles, whereas DC electrical conduction originates from small polarons translation [30]. On the basis of dielectric measurements performed in wide temperature range, and analyses of the behavior of different parameters (*s*, *W_H_*, *τ_M”_* and *σ_DC_*), it was confirmed that the electrical conductivity of Fe_3_O_4_ nanoparticles is related to the two different mechanisms (correlated barrier hopping and non-overlapping small polaron tunneling mechanisms), and that the transition between them is smooth.

## 2. Results and Discussion

### 2.1. Structure Analysis

**Figure 1:**
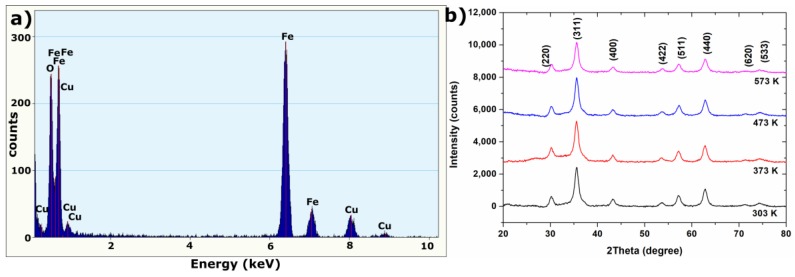
(**a**) EDS spectrum of synthesized Fe_3_O_4_ nanoparticles and (**b**) XRD patterns of Fe_3_O_4_ NPs recorded at different temperatures with marked Miller indices.

Figure 1a presents EDS spectrum of synthesized Fe_3_O_4_ nanoparticles. Observed peaks, associated with existence of iron and oxygen, confirm the presence of iron oxides in the synthesized material. The Cu peaks originate from the copper grid for EDS-TEM measurement. In order to determine the structure of the synthesized sample, XRD patterns were collected for the obtained powder, and are presented in Figure 1b. A magnetite structure (space groups: *Fd-3m*, DB card number: 9002318) was observed. Moreover, no phase transition (even at 573 K) was observed during the heating of the sample in the air.

The crystallinity volume size of magnetite nanoparticles was calculated based on Debye—Scherrer formula:

(1) $$D=\frac{K\lambda }{\beta cos\theta }$$

where *D* is the average crystallites size, *K* is the shape factor (for magnetite equaled 0.94), *λ* is the X-ray wavelength, and *β* is the full width at half-maximum of the highest intensity reflection (311) at diffraction angle *θ*. Obtained results are presented in Table 1. In this temperature range, no significant changes in crystallite size were observed.

**Figure 2:**
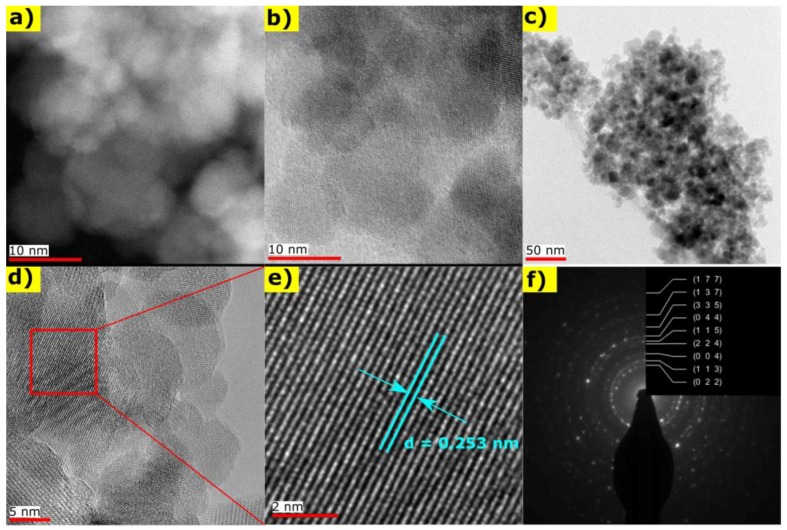
The STEM and the TEM images of Fe_3_O_4_ nanoparticles: (**a**) the HAADF STEM image and corresponding the BF/DF STEM image presented on (**b**); (**c**) the TEM image of highly agglomerated structure of Fe_3_O_4_ nanoparticles; (**d**) the HRTEM image of nanoparticles and lattice spacing between the (311) lattice planes identified on red marked area and visualized on figure (**e**); (**f**) the SAED pattern with marked Miller indices for Fe_3_O_4_ crystalline structure.

The structure and morphology of synthesized Fe_3_O_4_ NPs were analyzed on STEM and TEM images presented in Figure 2a–e. The crystalline structure of nanoparticles was visible on HAADF and BF/DF STEM images (Figure 2a,b). A highly agglomerated structure is present on the TEM image in Figure 2c. The lattice spacing between the (311) lattice planes in Fe_3_O_4_ was identified in Figure 2d, and marked on Figure 2e. Additionally, an SAED pattern with marked Miller indices for magnetite is presented in Figure 2f; this confirmed the phase purity of synthesized nanoparticles. The obtained results were compared in Table 2 with the analysis of XRD pattern at 303 K. The lattice spacing derived from XRD and SAED patterns confirms the existence of a magnetite structure, whereas the results obtained from XRD pattern are closer to the theoretical values.

### 2.2. Dielectric Properties

**Figure 3:**
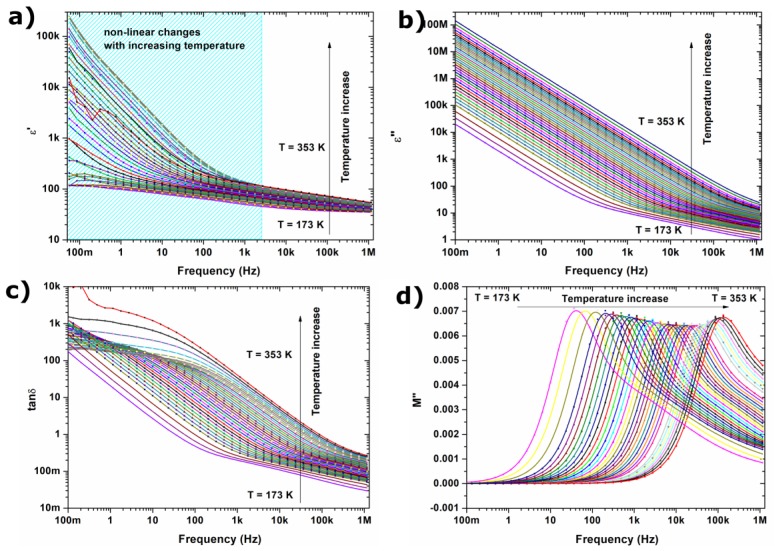
Variation of (**a**) real part of permittivity; (**b**) imaginary part of permittivity; (**c**) *tanδ* and (**d**) imaginary part of electric modulus *M”* with frequency at wide temperature range 173–353 K (temperature step was equaled 10 K for 173–203 K and 5 K for 203–353 K temperature range).

Figure 3a,b presents real (*ε’*) and imaginary (*ε”*) parts of complex permittivity. The dielectric spectra are dominated by a polarization process, which originates from the grain boundaries of ferrite nanoparticles. The value of the dielectric constant is inversely proportional to the grain boundary thickness i.e., thin grain boundaries are responsible for high values of electric permittivity [31]. The observed Maxwell-interfacial polarization [32] is related to the electron hopping in lower frequency region; this is discussed later in this study and in literature [33]. Borhan et al. described the role of grain boundaries in electron hopping in Zn-doped lithium ferrites. In the low-frequency region, charge carriers accumulate in the grain boundaries, and the hopping process requires more energy; therefore, the value of the imaginary part of the permittivity is very high [33]. Additionally, nonlinear changes in the value of dielectric loss tangent (*tanδ*) were observed for the low frequency region. The *tanδ* presented in Figure 3c is very high; this can be associated with the leaky capacitive nature of the sample.

The frequency dependence of imaginary part of electric modulus *M”* at different temperatures was shown in Figure 3d. The *M”* electric modulus peak is associated with the conduction relaxation processes; it can be expressed by the equation:

(2) $$M\prime \prime =i\;tan\delta /\varepsilon \prime \left(1+tan^{2}\delta \right)$$

It can be observed that at low temperatures, two different processes are visible, as the *M”* peak has a characteristic excess wing on the high frequency shoulder. These two processes, with different activation energies, merge at high temperatures. The slower process can be identified as a hopping of the charge carrier, while the visible excess wing (faster process) can be associated with the confinement of ions in their potential well, and localized motion of them—short distance mobility [34,35]. Therefore, the peak observed in modulus representation can be associated with a transition between long and short range mobility and the temperature dependent hopping mechanism in tested material [36].

### 2.3. Electrical Conduction Mechanism

**Figure 4:**
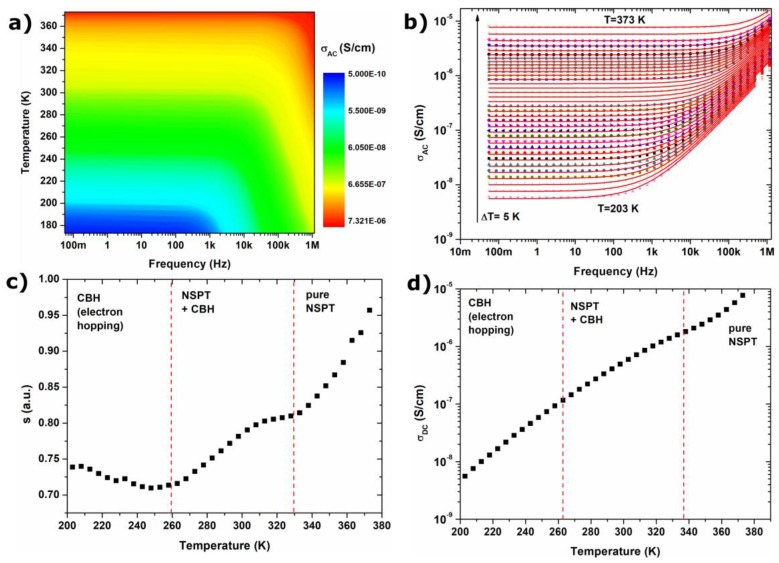
Electrical conductivity in compressed sample of Fe_3_O_4_ NPs: (**a**) 2D surface plot of AC conductivity in function of temperature and frequency; (**b**) AC conductivity measured for Fe_3_O_4_ NPs with solid line corresponding to the fit according to Jonscher’s power law; (**c**) *s*(*T*) behavior characteristic for two different CBH and NSPT models at different temperature ranges; (**d**) DC conductivity depending on temperature, calculated based on Jonscher’s power law.

Figure 4 shows the frequency and temperature dependence of AC conductivity of compressed Fe_3_O_4_ NPs. The conductivity increases with increasing frequency, which is characteristic for disordered solids, oxides and nanocomposites. The frequency dependence of electrical conductivity in ferrites can be described by Koop’s theory. In Koop’s theory, conductivity exhibits dispersion in higher frequencies (*f* > 10^4^ Hz for room temperature), which is associated with the existence of grains with high conductivity and their boundaries exhibiting high resistance [37]. In the high frequency region, the increase in conductivity can be explained by the intensified hopping of charge carrier phenomenon. An increase in the value of AC and DC conductivity can be also associated with the increase of probability of charge carriers tunneling, which is related to the thermal oscillations of sites [38]. Additionally, the AC conductivity follow to the Jonscher’s power law given by [39]:

(3) $$\sigma_{AC}=A{\left(2\pi f\right)}^{s}+\sigma_{DC}$$

where *A* is the characteristic parameter, *f* is the frequency and *s* is the exponent dependent on temperature and frequency with values in the range from 0 for ideal ionic-type crystals and to 1 for ideal Debye dielectric dipolar-type crystals. The value of the exponent *s* in the range 0 < *s* < 1 is characteristic for hopping conduction phenomena [23]. Basing on the *s*(*T*) relation, the conduction mechanism under the applied AC field can be determined. The function of *s* versus temperature (presented in Figure 4c) is characteristic for hopping mechanism (1 < *s* < 0). This conductivity mechanism is associated with the existence of Fe^3+^ and Fe^2+^ ions in magnetite structure, and in the literature it has been attributed to electron hopping between them [40,41]. In ferrites, the charge carriers are localized at the magnetic ions, and all Fe^2+^ ions in the octahedral site participate in the hopping transport. The hopping mechanism of conductivity was confirmed by analysis of the *s*(*T*) behavior. In a low temperature region, a decrease in the *s* value was observed, which can be associated with the CBH model. In this model, conduction is associated with charge carriers hopping process [25,39]. With increasing temperatures, the plateau region appears even at higher frequencies, and *s*(*T*) had an increasing tendency. According to the Koop’s theory, this region is associated with the resistance of the grain boundary. With increasing temperatures, grain boundary resistance changes the conduction mechanism, and two models, CBH and NSPT, can be applied in order to describe the conduction mechanism. For the highest temperature region, only the NSPT type model can be applied to describe the *s*(*T*) behavior. In this model, small polarons are formed when a charge carrier (as moving electrons in lattice) deforms the surrounding lattice, and a tunneling process is responsible for conductivity.

The higher conductivity and plateau region observed even for higher frequencies are associated with higher electron mobility at higher temperatures. The electron mobility can be calculated based on the Equation (4), in which *σ_DC_* is the DC electrical conductivity obtained from Jonscher’s power law at different temperatures, *M* is the molecular weight of magnetite, *ρ* is the bulk density of magnetite (5.18 g/cm^3^), *n_Fe_* is the number of iron in chemical formula, *e* is the charge of the carriers, and *N_A_* is the Avogadro constant [30]:

(4) $$\mu =\frac{\sigma_{DC}M}{N_{A}\rho n_{Fe}e}$$

**Figure 5:**
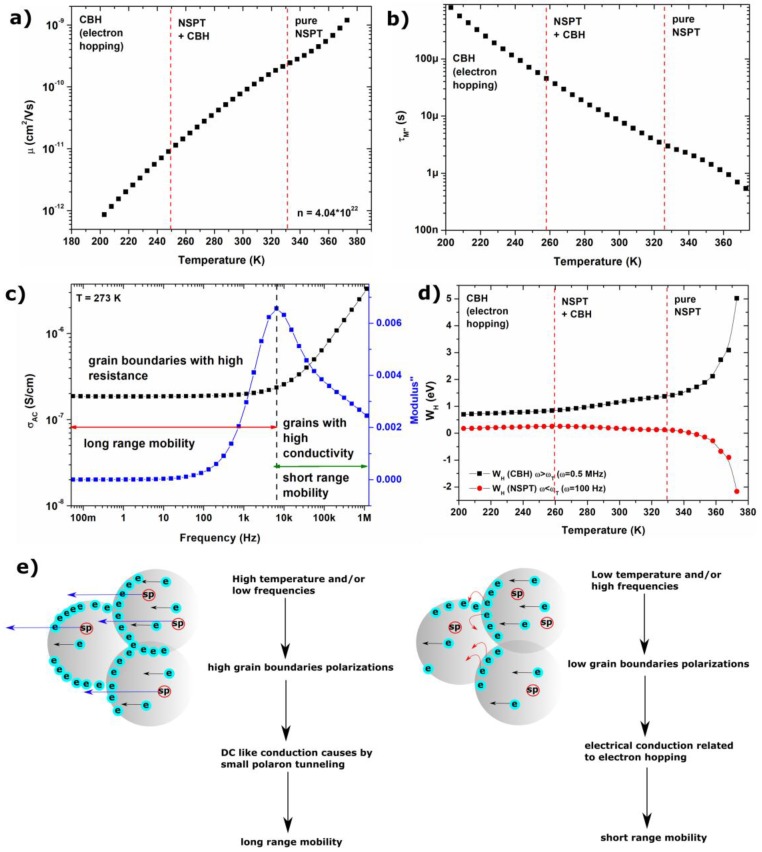
(**a**) electrons mobility in Fe_3_O_4_ NPs; (**b**) relaxation times calculated for *M”* peak associated with the transition between long and short mobility; (**c**) summarizing of analyses of the dielectric properties and AC conductivity, prepared for 273 K, with marked regions related to the grain boundaries and grain conductivity, and corresponding to them long and short range mobility with the border corresponding to the maximum *M”* peak; (**d**) the value of hopping energy, calculated based on CBH and NSPT models for two circular frequencies—100 Hz and 0.5 MHz; (**e**) schematic representation of two conduction mechanism identified in different temperature and frequency regions: the blue arrow represents tunneling of small polarons, the red solid arrow electron hopping, and the black arrow electrons moving in crystal structure between Fe^3+^ and Fe^2+^ ions.

The obtained results have been presented in Figure 5a. It can be seen that with increasing temperatures, the mobility of electrons increases; their number is equal to 4.04 × 10^22^. The *M”* peak (see Figure 3 for details) can be associated with the transition between long and short range mobility, for which characteristic relaxation time can be calculated by applying formula: *τ_M”_* = 1/2*πf*. The function of relaxation time versus temperature has been presented in Figure 5b. The obtained decreasing tendency of relaxation times is characteristic of different materials, and has been identified among other ferrites. Moreover, observed changes are not linear over wide temperature ranges, so the previously postulated different conduction mechanism in low and high temperature region can be confirmed, as well as the smooth transition between them.

In CBH and NSPT model value of *s* exponent can be calculated based on Equations (5) and (6) respectively [42,43,44].

(5) $$s=1-\frac{6kT}{W_{H}-kTln\left(\frac{1}{\omega \tau_{0}}\right)}$$

(6) $$s=1-\frac{4}{ln\left(\frac{1}{\omega \tau_{0}}\right)-\frac{W_{H}}{kT}}$$

where *W_H_* is the hopping energy, *k* is the Boltzmann constant, *T* is the temperature, *ω* is the circular frequency (*ω* = 2*πf*), and *τ*_0_ is the characteristic relaxation time equaled to 10^−13^ s.

According to the Koop’s theory and *s*(*T*) behavior, the value of hopping energy for electron hopping and small polaron tunneling can be calculated for different temperatures from relationships between *W_H_* and *s*. The relaxation time corresponding to the transition frequency between long and short range mobility (schematically presented for 273 K on Figure 5c) value of *W_H_* can be calculated for small polaron tunneling (for frequencies below transition frequency) and for electron hopping (for frequencies above transition frequency). In Figure 5d *W_H_*(*T*) for *ω* = 100 Hz and *ω* = 0.5 MHz were presented. It can be noticed that with increasing temperatures, the value of *W_H_* for electron hopping increases, whereas *W_H_* for small polaron tunneling decreases. Therefore, in higher temperature regions, small polaron tunneling is a privileged process and occurs spontaneously (*W_H_* < 0 eV) due to higher internal energy of particles.

In summary, in Figure 5e conduction mechanisms identified in compressed Fe_3_O_4_ nanoparticles are presented schematically. At high temperatures and low frequencies, tunneling of small polarons occurs, which is associated with the polarization of grain boundaries, and manifests itself as long-range mobility. For high frequencies and low temperatures, electron hopping is the main conduction mechanism, what is associated with CBH model and short-range mobility.

## 3. Materials and Methods

Magnetite NPs were synthesized by the simple co-precipitation method, using polyvinylpyrrolidone (PVP) as surfactant. For this purpose, 100 cm^3^ solution of FeCl_3_ and FeSO_4_ (with molar proportion of 2:1) was prepared. Next, 5 g of PVP and 5 cm^3^ of HCl were added and the solution was heated to 55 °C in an ultrasonic bath. Afterwards, a 50 cm^3^ solution containing 3.2 g NaOH was added dropwise to the prepared solution containing iron ions. Additionally, to complete the reaction, 20 cm^3^ of solution containing 2 g NaOH was also added dropwise, and a sonication process was carried out for an additional 10 min. The synthesized nanoparticles were filtered, and washed with water and ethanol. The black powder was then dried at 40 °C. The obtained nanoparticles were next compressed using a hydraulic press to form them into discs, in order to measure dielectric properties and electrical conductivity under an AC field. The samples, with a diameter of 10 mm and thickness of 0.895 mm, were obtained using a compression pressure of 30 bar. Energy-dispersive X-ray spectra, electron microscope images, and selected area electron diffraction (SAED) patterns were obtained using a transmission electron microscope S/TEM TITAN 80-300 (FEI Company, Eindhoven, The Netherlands). For this purpose, Fe_3_O_4_ NPs were dispersed in ethanol and sonicated. Two drops of this dispersion were placed into the copper grid with a carbon film, and dried at ambient temperature. The SAED patterns were analyzed using a diffract GUI tool (CrystBox software, version 1.10 (build 0066), Institute of Physics of the Czech Academy of Sciences, Prague, Czech Republic [45,46]). X-ray diffraction patterns at different temperatures were collected using X-ray diffractometer Rigaku MiniFlex 600 with a copper tube Cu Kα (*λ* = 0.15406 nm), a tube voltage of 40 kV, and a current of 15 mA, using a D/teX Ultra silicon strip detector and the BTS 500 high temperature attachment (Rigaku Corporation, Tokyo, Japan). Dielectric measurements were performed using the Concept 81 dielectric spectrometer, equipped with an Alpha analyzer and the Novo-cool temperature control system (Novocontrol, Montabaur, Germany). The compressed samples were placed in acid-resistant steel capacitors with a diameter of 20 mm. Measurements were carried out in the frequency range of 10^−2^–10^6^ Hz, and a temperature range from 173 K to 373 K.

## 4. Conclusions

The analysis of the behavior of different parameters (*s*, *W_H_*, *τ_M”_* and *σ_DC_*) confirms that the electrical conductivity of Fe_3_O_4_ nanoparticles is related to two different mechanisms, and that the transition between them is smooth. The most visible changes can be observed during analyses of the behavior of *s*(*T*) and *W_H_*(*T*). Moreover, in this study, comprehensive analyses were used to describe electrical conductivity in different inhomogeneous materials. It was noted that the analysis of the behavior of *W_H_*(*T*) can be used to determine the transition between different conduction mechanisms. However, an analysis needs to be made at a broad temperature and frequency range, separately. Additionally, on the basis of presented analysis one can observe that:

1. Electrical conductivity of agglomerated and compressed Fe_3_O_4_ nanoparticles is associated with the structure of the sample, and can be described by Maxwell-Wagner model and Koop’s theory.
2. In the low frequency region, electrical conductivity is associated with long-range mobility and grain boundaries with high resistance, whereas in high frequencies region, it is related to the short-range mobility, and grains with high conductivity.
3. With increasing temperature, conductivity related to the grain boundaries can be observed within a wider frequency region, which is associated with the shift of peaks related to the electrical relaxation process.
4. The maximum of the peaks observed on *M”*(*f*) plots is associated with a transition between long and short range mobility.
5. Increasing the value of *ε’* and *ε”* in the low frequency region is associated with grain boundaries polarization, according to the Maxwell-Wagner model; in the low-frequency region, charge carriers accumulate in grain boundaries, and the hopping process requires more energy.
6. AC conductivity in Fe_3_O_4_ nanoparticles follows the Jonscher’s power law, characteristic for disordered solids; electrical conductivity is associated with two mechanisms described by correlated barrier hopping and non-overlapping small polaron tunneling models.
7. CBH and NSPT models are valid for different temperature and frequency regions. For low temperatures and high frequencies, the conduction mechanism can be described by the CBH model, whereas for high temperatures and low frequencies, the NSPT model is more adequate.
8. The value of hopping energy for the tunneling of small polarons decreases with increasing temperatures, and in higher temperatures *W_H_* < 0 eV; thus tunneling occurs spontaneously.

## Figures and Tables

**Table 1:** The average crystallite volume size calculated for Fe_3_O_4_ NPs at different temperatures.

*T* (K)	*2θ* (degree)	*D* (nm)
303	35.55	9.01
373	35.53	8.89
473	35.56	9.07
573	35.57	9.44

**Table 2:** Lattice spacing distance for different lattice planes obtained from X-ray and TEM diffraction patterns.

Plane	Theoretical d-Spacing (nm)	d-Spacing (nm) (SAED Pattern)	d-Spacing (nm) (XRD at 303 K)
220	0.296	0.296	0.296
311	0.253	0.257	0.252
400	0.210	0.213	0.209
422	0.171	0.172	0.171
511	0.161	0.163	0.161
440	0.148	0.149	0.148
533	0.128	0.129	0.128
